# Influence of Specialization from Intensive Care Physicians on Outcome in Multiply Injured Patients—A Matched-Pair Analysis at a Level I Trauma Center

**DOI:** 10.3390/life15091407

**Published:** 2025-09-06

**Authors:** Gökmen Aktas, Larissa Rolfes, Maximilian Koblenzer, Vesta Brauckmann, Jorge Mayor, Jan Clausen, Jonas Ajouri, Tarek Omar Pacha, Stephan Sehmisch, Philipp Mommsen

**Affiliations:** 1 Department of Trauma Surgery, Hannover Medical School, Carl-Neuberg St. 1, 30625 Hannover, Germany; rolfes.larissa@mh-hannover.de (L.R.); koblenzer.maximilian@mh-hannover.de (M.K.); brauckmann.vesta@mh-hannover.de (V.B.); mayorramirez.jorge@mh-hannover.de (J.M.); clausen.jan-dierk@mh-hannover.de (J.C.); omarpacha.tarek@mh-hannover.de (T.O.P.); sehmisch.stephan@mh-hannover.de (S.S.); mommsen.philipp@mh-hannover.de (P.M.); 2 Department of Anaesthesiology and Critical Care, Campus Kassel of the Philipps University Marburg, Möncheberg St. 41-43, 34125 Kassel, Germany; ajouri.jonas@gnh.net

**Keywords:** critical care, intensive care, trauma, polytrauma, trauma surgery, anesthesiology, intensive care unit

## Abstract

The specialty background of intensive physicians managing severely injured patients varies internationally, with trauma ICUs often led by either trauma surgeons or anesthesiologists, both of whom receive additional intensive care training. Whether physician specialty affects outcomes remains uncertain. We conducted a retrospective single-center cohort study of patients aged ≥ 16 years with an Injury Severity Score (ISS) ≥ 16 admitted to a level I trauma center between January 2005 and December 2022. Patients were treated either in a trauma surgery ICU (T-ICU) or an anesthesiology ICU (A-ICU). Briefly, 1:1 matching was conducted based on demographic and injury-related variables, with the primary outcome being in-hospital mortality and secondary outcomes including transfusion requirements, duration of mechanical ventilation, ICU and hospital length of stay, and Glasgow Outcome Scale (GOS) at discharge. Among the 1015 eligible patients (T-ICU: n = 920; A-ICU: n = 95), 52 patients (26 per group) were successfully matched with comparable baseline characteristics. No significant differences were observed in mortality, GOS, transfusion requirements, ventilation duration, or ICU/hospital length of stay. These findings suggest that, when both are led by certified intensive care specialists, trauma surgery- and anesthesiology-based ICUs achieve comparable outcomes, supporting multidisciplinary models while highlighting the need for larger multicenter studies.

## 1. Background

The care of severely injured patients presents a challenge for prehospital and hospital treatment, requiring interdisciplinary expertise specialized in trauma management and critical care [1]. Intensive care medicine is a relatively young and highly technologized field with steadily increasing importance in clinical patient care [2,3]. The specialized knowledge required and the high demands placed on quality of care for patients in intensive care units can best be met by a highly specialized intensive care team working closely with the primary treating physicians [2].

According to the German Interdisciplinary Association for Intensive Care and Emergency Medicine (DIVI), an intensive care unit should be led by a physician who holds an additional qualification in critical care medicine [4]. To acquire this qualification in Germany, physicians complete residency training in specialties such as anesthesiology, surgery, internal medicine, pediatrics, neurosurgery, or neurology, followed by an 18-month training in critical care medicine at a certified facility. There is no specific recommendation regarding which discipline is particularly suited for critical care medicine [5].

The trauma intensive care unit at Hannover Medical School is run solely by surgeons. A leading surgical intensivist is on-site daily. The resident doctors complete their rotation in the ICU for 6 to 12 months after finishing their training in the emergency department and on the general ward. After completing the rotation, they continue to provide on-call services in the ICU for an additional 12–24 months. Additionally, a surgical intensivist is available daily for consultations and can be called in for urgent cases. Every resident doctor must complete this rotation during their training. This ensures that every senior surgical physician has knowledge of intensive care in their future career and can contribute to the care of severely injured patients, supporting their intensive care treatment. The anesthesiology residents follow a typical training pathway leading to an ICU rotation. After short periods in one of the intensive care units, a one-year ICU rotation is scheduled in their fourth year of training. The Department of Anesthesiology provides intensive care beds for all departments, allowing residents to gain a broad spectrum of experience in intensive care medicine. In both ICUs, patient care is provided by state-certified specialist nurses in intensive and anesthesia care, each having completed a two-year advanced training program.

In the USA, training to become an intensivist is based on the curriculum of the Accreditation Council for Graduate Medical Education (ACGME) [6]. Similarly to Germany, a completed residency in specialties such as, anesthesiology, internal medicine, or surgery is required for a fellowship in critical care [6]. However, unlike in Germany, the fellowship is not uniform but tailored to each specialty [7,8], following either the ACGME Anesthesiology Critical Care Medicine or ACGME Surgical Critical Care guidelines. Additionally, standardized training in Advanced Trauma Life Support (ATLS), Advanced Cardiac Life Support (ACLS), and Intensive Life Support (ILS) has become increasingly important for physicians working in trauma intensive care settings, regardless of their primary specialty.

Anesthesiology has increasingly become prominent in perioperative, preclinical and clinical emergency management, intensive care medicine, and pain therapy [9]. Anesthesiologists recognized the importance of intensive care medicine early and dedicated themselves to it as an emerging discipline since the 1950s, while in surgery, intensive care medicine was one area among many [10,11]. According to the European Society of Intensive Care Medicine (ESICM), anesthesiologists provide more than half of the intensive care in Europe [6]. Intensivists with specialized training have proven beneficial for critically ill patient survival in many studies [12].

In contrast, trauma surgeons with specialized training in surgical intensive care can achieve positive outcomes through consistent care under unified management, starting from the trauma room and emergency surgeries to subsequent intensive care and rehabilitation [13]. The early recognition of posttraumatic and postoperative complications is vital, and addressing these complications is often the responsibility of surgeons experienced in intensive care medicine [12].

Intensive care medicine has always been integral to surgery, and its significance remains undiminished [14]. However, the growing distinction of intensive care within anesthesiology compared to surgically led intensive care highlights the need to explore potential differences between these specialties in the care of critically injured patients.

The primary aim of this study was to assess whether the primary specialty background of the attending intensive care physician (trauma surgery vs. anesthesiology) influences patient outcomes in multiply injured patients. The primary outcome measure was in-hospital mortality. The secondary aims were to compare treatment-related parameters between the two ICU settings, including transfusion requirements (PRBC, FFP, platelets), duration of mechanical ventilation, ICU length of stay, total hospital length of stay, and functional outcome at discharge (Glasgow Outcome Scale).

## 2. Patients and Methods

In a retrospective single-center cohort study, all multiple trauma patients admitted to our level I trauma center between January 2005 and December 2022 were enrolled.

### 2.1. Inclusion and Exclusion Criteria

Multiple injured patients with an Injury Severity Score (ISS) ≥ 16, a threshold historically widely used to define polytrauma, and a minimum age of 16 years primarily admitted to our hospital and treated in an intensive care unit run by trauma surgeons (T-ICU group) or by anesthetists (A-ICU group) were included. Patients who were secondarily transferred or died before hospital or intensive care unit admission were excluded.

### 2.2. Patient Allocation Process

The Department of Trauma Surgery leads the shock room in the emergency department. Since our Level 1 Trauma Center primarily maintains a trauma surgery ICU, polytrauma patients are preferentially transferred to the trauma ICU. When the trauma ICU reaches capacity, severely injured patients are admitted to the anesthesiology ICU. This allocation procedure was maintained consistently throughout the study period but may have introduced potential selection bias, which we addressed through the rigorous matching procedures described below.

### 2.3. Data Collection and Parameters

Demographic and baseline data including age, sex, and ASA score (classification of patients regarding their physical condition, American Society of Anesthesiologists) before the accident, duration of mechanical ventilation, and length of intensive care and overall inpatient stay, as well as transfusion requirements (packed red blood cells, fresh frozen plasma, and platelet concentrates), were acquired from the hospital information system. The injury pattern regarding different body regions (head, face, chest, abdomen, extremities, external) was evaluated using the 2008 update of the Abbreviated Injury Scale (AIS), and overall injury severity was calculated according to the Injury Severity Score (ISS). Systolic blood pressure and heart rate at admission were collected for calculating the shock index (SI = HR/SBP), with shock defined as a shock index > 1.0.

### 2.4. Outcome Measures

The primary outcome measure of this study was in-hospital mortality. Secondary outcome measures included transfusion requirements within the first 48 h and throughout the entire hospital stay, comprising packed red blood cells, fresh frozen plasma, and platelet concentrates. Additional secondary outcomes were the duration of mechanical ventilation, ICU length of stay, total hospital length of stay, and the Glasgow Outcome Scale at discharge. These measures were chosen for their clinical relevance and because they were reliably available in our retrospective dataset.

### 2.5. Statistical Analysis

Descriptive analyses were performed for the entire study population as well as for the trauma surgery (T-ICU) and anesthesiology group (A-ICU). For normally distributed data, parametric tests (*t*-test) were used. Non-normally distributed data were analyzed with non-parametric testing methods (Mann–Whitney test for independent variables, Wilcoxon test for dependent variables). The chi-square test was utilized in the analysis of contingency tables.

To minimize potential selection bias and confounding factors, we performed a 1:1 matching approach rather than propensity score matching. Matching criteria included age, gender, ASA score, injury patterns (AIS scores), and overall injury severity (ISS). For each pair, we matched one patient from the A-ICU group with a corresponding patient from the T-ICU group. We used a tolerance deviation of 0.1 for continuous variables (age, ASA, AIS scores, and ISS), meaning that the maximum allowed difference in these parameters was kept within 0.1 standard deviations of the respective parameter, which represents a compromise between reducing bias and maintaining adequate sample size. Categorical variables like gender were exactly matched.

A binary logistic regression analysis was conducted with the calculation of adjusted Odds Ratios (OR) and 95% Confidence Intervals (95% CI) for mortality, controlling for age and ISS.

Statistical analysis was performed using IBM SPSS computer software (SPSS 28, IBM, Armonk, NY, USA). Statistical significance was set at a *p*-value < 0.05.

## 3. Results

**Figure 1:**
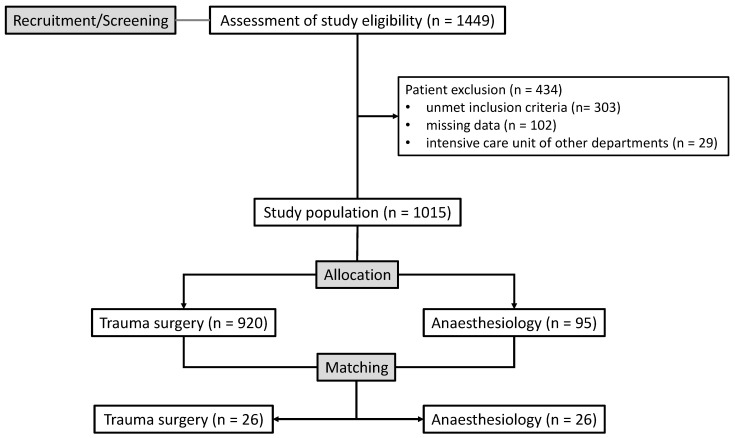
Study patients’ flow diagram.

Overall, 1449 patients were recruited and assessed for study eligibility. A total of 434 patients were excluded due to non-fulfillment of inclusion criteria, missing data and treatment on an intensive care unit other than traumatology or anesthesiology (Figure 1). Before matching, several differences were observed between the groups. Patients admitted to the A-ICU tended to have worse pre-existing medical conditions (ASA 1.6 ± 0.7 vs. 1.4 ± 0.7, *p* = 0.044), while those in the T-ICU had more severe extremity and pelvic injuries (AIS 2.3 ± 1.4 vs. 1.8 ± 1.4, *p* = 0.006). T-ICU patients more frequently presented with shock at admission (18.9% vs. 10.5%, *p* = 0.043) and received higher volumes of packed red blood cells (10.4 ± 16.3 vs. 7.5 ± 11.3 units, *p* = 0.025) and platelet concentrates (1.2 ± 3.4 vs. 0.7 ± 1.4 units, *p* = 0.008). This pattern of patient allocation suggests that the T-ICU preferentially received patients with more severe extremity trauma requiring greater transfusion volumes, while the A-ICU received patients with poorer baseline health status.

After 1:1 matching, 52 patients remained, equally distributed (n = 26) between the T-ICU and A-ICU groups. Demographic, clinical, and outcome data for the matched population are summarized in Table 1. The quality of matching was excellent, with no significant differences in age, gender distribution, pre-existing medical conditions, injury patterns, or injury severity between groups. Additionally, there were no differences in the presence of shock at the time of admission between the matched groups.

Treatment parameters regarding duration of mechanical ventilation, intensive care stay, and in-hospital stay were between the matched T-ICU and A-ICU group. Transfusion requirements for PRBC, FFP and PC within the first 48 h and throughout the entire hospital stay were also not different.

The mortality in the matched overall population was 13.5% (n = 7). In the matched T-ICU group, 2 patients died (7.7%), compared with 5 patients in the A-ICU group (19.2%), with no statistically significant difference (*p* = 0.223). Our logistic regression analysis, controlling for age and ISS, yielded an adjusted odds ratio for mortality (A-ICU vs. T-ICU) of 2.84 (95% CI: 0.49–16.51, *p* = 0.245). While this suggests a trend toward higher mortality in the A-ICU group, the wide confidence interval crossing 1 indicates that this difference could be due to chance. No differences were also found concerning the Glasgow Outcome Scale (GOS).

## 4. Discussion

The aim of this study was to analyze the influence of the intensive care medical specialty (trauma surgery, anesthesiology) on the outcome of severely injured patients. A total of 1015 severely injured patients were included, of whom the vast majority (90.6%) were treated in the trauma surgery intensive care unit. Compared to patients treated in the anesthesiology intensive care unit, the trauma surgery patient cohort had more severe extremity and pelvic injuries as measured by the AIS. Increased blood loss associated with severe extremity and pelvic injuries may explain why patients in the trauma surgery intensive care unit were significantly more often in shock at the time of admission, and the need for transfusion of erythrocyte and platelet concentrates was significantly higher. Despite this, there was a statistical trend (*p* < 0.1) towards a lower mortality rate in the trauma surgery patient group compared to patients treated in the anesthesiology intensive care before matched-pair analysis. Possible reasons for this trend could be the slightly worse pre-traumatic health status as measured by the ASA score and the more severe head injuries in patients treated in the anesthesiology intensive care unit. The mortality rate of the overall population of 11.4% described in our study is consistent with the current literature.

The observed differences in the distribution of severely injured patients, including the higher proportion of cases with extremity and pelvic injuries in the trauma surgery intensive care unit, are primarily the result of bed availability rather than intentional selection. As outlined in the Methods section, the trauma surgery ICU serves as the primary receiving unit for polytrauma patients whenever capacity is available, whereas the anesthesiology ICU admits patients only when the trauma surgery ICU has reached full capacity. This allocation procedure, which was applied consistently throughout the study period, was driven solely by logistical considerations and not by predefined injury patterns or specialty-specific admission criteria.

To counteract the potentially biasing influence of patient assignment based on the injury pattern, a matched-pair analysis was conducted with matching factors of age, gender, ASA, AIS, and ISS. Our matching was conducted using a 1:1 approach with a tolerance deviation of 0.1 for continuous variables (age, ASA, AIS scores, and ISS). This means that for each matched pair, the maximum allowed difference in these parameters was kept within 0.1 standard deviations, ensuring high comparability between groups. The quality of this matching is confirmed by our validation analyses, which showed no significant differences between the matched groups in any of these parameters (all *p*-values > 0.05). While propensity score matching was considered because of its ability to combine several covariates into a single score and allow more flexible matching criteria, its potential advantage in sample size was limited in our dataset by the smaller size of the A-ICU group (n = 95 vs. 920 in T-ICU). Even under optimal PSM conditions, the achievable maximum sample size of 95 patients would have only marginally exceeded our final matched cohort of 52 patients and would not have substantially improved the statistical power to detect small effects.

In the matched patient cohort, younger patients (19.8 ± 2.6 vs. 45.4 ± 19.7 years) with a higher ISS (32.5 ± 10.1 vs. 28.6 ± 10.2) were found compared to the overall study population. A significant influence of the intensive care medical specialty on treatment and outcome in severely injured patients could not be demonstrated (mortality (*p* = 0.223) and length of the overall stay (*p* = 0.288)). Our logistic regression analysis, after controlling for age and ISS, yielded an adjusted odds ratio for mortality (A-ICU vs. T-ICU) of 2.84 (95% CI: 0.49–16.51, *p* = 0.245). While this suggests a trend toward higher mortality in the A-ICU group, the wide confidence interval crossing 1 indicates that this difference could be due to chance. This suggests that the quality of care in both specialties appears to be comparable, and the choice of the medical specialty of the leading intensive care unit (trauma surgery, anesthesiology) apparently does not play a decisive role in the overall treatment outcome of severely injured patients.

Our findings are consistent with those of the study by Lee et al., who reported no difference in mortality in a mixed medical–surgical–trauma ICU, regardless of whether critically ill patients were managed by internists, surgeons, or anesthesiologists [15]. Similarly, earlier work demonstrated that for various critical illnesses, mortality did not differ between patients admitted to general ICUs and those treated in specialized ICUs [16]. In contrast, for intracranial hemorrhage, there is some evidence of a survival benefit when patients are cared for in a dedicated neurosurgical ICU [17]. For polytraumatized patients, however, the available data remain very limited. In the subgroup of trauma patients with traumatic brain injury, outcomes appear to be more favorable when they are treated in trauma ICUs compared with neuro-ICUs or mixed medical–surgical ICUs [18]. In addition, Park et al. demonstrated that specialized trauma ICUs were associated with shorter ventilation times and reduced ICU and hospital length of stay, and therefore may also be more cost-effective [13]. Our study found no significant differences in these parameters between treatments by anesthesiologists or trauma surgeons. One possible explanation is the progress in training over the last two decades. The study by Park et al. was conducted almost 25 years ago. Since then, intensive care education has become more standardized across all specialties involved in trauma care. Courses such as ATLS and ACLS have been widely implemented. Structured curricula now ensure that both anesthesiologists and surgeons receive comparable training in critical care.

The care of polytrauma patients is highly complex. Effective treatment of severely injured patients involves a combination of organ-supportive measures, including ventilation, hemodynamic stabilization, and infection management, together with a careful evaluation of traumatic injuries and the necessary surgical management at the appropriate time [14]. Anesthesiologists contribute particular expertise in pain control, maintaining vital functions and managing emergencies [6,12]. Trauma surgeons, on the other hand, provide operative expertise, including the evaluation of complex injuries, the timing of surgical interventions, and the recognition of complications such as compartment syndrome or uncontrolled bleeding [19,20]. Therefore, multidisciplinary collaboration may be more important than strict specialty-based leadership. Our data support this assumption, as no outcome differences were found between the two groups when standardized protocols were applied.

In times of staff shortages, and rising patient numbers intensive care units remain a critical resource [3,21]. To improve outcomes and reduce costs, working groups of the National Institutes of Health, the Society of Critical Care Medicine, and the American College of Chest Physicians recommend regionalization of intensive care medicine [22]. This has already led to interdisciplinary surgical ICUs, where patients from various surgical disciplines are treated together. Such integration reduces the need for multiple specialty-specific ICUs and are particularly relevant for smaller regional hospitals. Schreiter and Saeger emphasized that merging specialty-specific ICUs into larger interdisciplinary units can provide economic and educational advantages, provided they are led by experienced intensive care physicians from either anesthesia or surgery [23]. Similarly, Neuhaus highlighted that close collaboration between these fields, rather than competition, is crucial for effective leadership in interdisciplinary ICUs [24].

Taken together, our findings suggest that the formal affiliation of an ICU to a specific department is less decisive than standardized treatment protocols, interdisciplinary collaboration, and continuous education of all involved specialists. For the care of severely injured patients, a multidisciplinary team approach may be more effective than specialty-based leadership models.

### Limitations

The present work shows some limits ought to be considered. Even though a high number of patients (n = 1015) could be included in the basic collective due to the retrospective study design and long investigation period, it was only possible to include 52 patients in the analysis for the 1:1 matching due to the “uneven distribution” and high number of matching factors, described above. The low number of cases represents one of the most significant limitations of the present study. Nonetheless, the matched study population represents a well-selected patient collective as a result of the strict matching criteria with an applied deviation tolerance of only 0.1. This ensures a high comparability of the two patient groups and consequently a high validity of the results.

While we attempted to address selection bias through our matching approach, we acknowledge that the original allocation of patients to T-ICU versus A-ICU was not randomized and was influenced by bed availability and potentially by clinical judgment regarding which specialty might be better suited for specific injury patterns. Our data show that before matching, patients in the A-ICU tended to have worse pre-existing medical conditions (higher ASA scores), while T-ICU patients had more severe extremity injuries and were more often in shock at admission. This pre-existing allocation bias was effectively addressed through our matching procedure, as confirmed by our validation analyses.

The limited sample size in our matched analysis affects the statistical power of our study. With 52 matched patients and observed mortality rates of 7.7% (2/26) in T-ICU versus 19.2% (5/26) in A-ICU, our study had limited power to detect statistically significant differences. Our analysis indicates that we would need an absolute difference of approximately 25% in mortality rates to achieve statistical significance (*p* < 0.05) with 80% power. This explains why the observed numerical difference (11.5%) did not reach statistical significance (*p* = 0.223), and suggests that smaller but potentially clinically important differences might have been missed due to sample size limitations.

Regarding outcome assessment beyond mortality and length of stay, we were unable to include comprehensive data on complications such as sepsis, ARDS, and multi-organ failure due to limitations in our retrospective data collection. The extended study period of 17 years resulted in inconsistent documentation of these complications, with different diagnostic criteria and documentation standards being used over time. Our database did not allow for reliable extraction of the temporal course of these complications, making it difficult to determine whether complications developed during ICU stay or were present upon admission. Instead, we focused on more reliably documented outcomes such as mortality and length of stay.

In addition to the small number of cases, the retrospective and monocentric study design as well as the long investigation period of 17 years represent further limitations of the present work. During this time, significant advances in trauma management occurred, including standardized treatment algorithms, improved diagnostics, and enhanced interdisciplinary collaboration. These developments likely improved overall outcomes and may have diminished potential specialty-specific differences. The lengthy study period makes it difficult to isolate the effects of medical specialty from evolving treatment standards, potentially limiting the validity and applicability of our findings to current clinical practice.

## 5. Conclusions

The first hours after a polytrauma are crucial for prognosis. Treatment requires a time- and priority-oriented concept. In the present study, no significant differences were found regarding the outcome of polytraumatized patients treated in ICUs led by trauma surgeons or anesthesiologists. Limitations include the small number of cases, the retrospective single-center design, and the long study period.

Our findings suggest that successful trauma care may depend more on multidisciplinary collaboration and standardized protocols than on the primary specialty of treating physicians. Both anesthesiologists and trauma surgeons can provide high-quality intensive care when adequately trained.

Further multicenter studies are needed to strengthen the validity and generalizability of these results. Future research should also explore outcomes of trauma patients managed in ICUs led by other specialties, such as neurosurgery or visceral surgery.

## Figures and Tables

**Table 1:** Demographic, clinical and outcome data for overall population, T-ICU and A-ICU group after matching.

Characteristics	Total (n = 52)	T-ICU (n = 26)	A-ICU (n = 26)	*p*-Value
Age (years), mean ± SD	19.8 ± 2.6	19.3 ± 1.6	20.2 ± 3.2	0.181
Male gender, n (%)	41 (78.8)	21 (80.8)	20 (76.9)	0.734
ASA, mean ± SD	1.1 ± 0.3	1.1 ± 0.3	1.2 ± 0.4	0.692
Injury Severity Score, mean ± SD	32.5 ± 10.1	31.9 ± 11.4	33.2 ± 8.9	0.666
Abbrevieated Injury Scale, mean ± SD Head Face Thorax Abdomen Extremities External	2.5 ± 1.7 0.9 ± 1.3 2.8 ± 1.5 1.4 ± 1.6 2.5 ± 1.2 0.9 ± 0.9	2.5 ± 1.6 0.6 ± 0.9 2.9 ± 1.4 1.7 ± 1.8 2.6 ± 1.1 1.0 ± 0.8	2.5 ± 1.8 1.2 ± 1.6 2.9 ± 1.6 1.2 ± 1.5 2.4 ± 1.3 0.9 ± 0.9	0.935 0.117 0.925 0.278 0.578 0.872
Shock, n (%)	10 (19.2)	6 (23.1)	4 (15.4)	0.482
Duration of mechanical ventilation (h), mean ± SD	185.0 ± 261.7	216.3 ± 262.6	153.0 ± 262.2	0.392
Duration of intensive care (days), mean ± SD	13.1 ± 15.9	15.3 ± 19.2	11.0 ± 11.7	0.33
Duration of hospital stay (days), mean ± SD	23.9 ± 24.4	27.5 ± 30.1	20.3 ± 16.6	0.288
Transfusion requirement PRBC 48 h (units), mean ± SD FFP 48 h (units), mean ± SD PC 48 h (units), mean ± SD PRBC total (units), mean ± SD FFP total (units), mean ± SD PC total (units), mean ± SD	6.7 ± 9.4 5.5 ± 8.0 1.0 ± 2.2 10.8 ± 14.0 7.9 ± 11.6 1.3 ± 2.4	7.5 ± 10.2 6.2 ± 9.3 1.4 ± 2.7 12.4 ± 14.8 9.7 ± 13.6 1.7 ± 3.1	5.9 ± 8.6 4.9 ± 6.6 0.7 ± 1.4 9.3 ± 13.3 6.2 ± 9.2 0.9 ± 1.5	0.522 0.563 0.235 0.441 0.294 0.239
Glasgow Outcome Scale, mean ± SD	3.9 ± 1.5	4.3 ± 1.1	3.6 ± 1.7	0.072
Mortality, n (%)	7 (13.5)	2 (7.7)	5 (19.2)	0.223

## Data Availability

The original contributions presented in this study are included in the article. Further inquiries can be directed to the corresponding author(s).

## References

[1] Haas N.P., Von Fournier C., Tempka A., Südkamp N.P. (1997). Trauma center 2000. How many and which trauma centers does Europe need around the year 2000?. Unfallchirurg.

[2] Burchardi H., Moerer O. (2003). Die ärztliche Versorgungsstruktur auf der Intensivstation: Internationale Erfahrungen. Intensivmed. + Notfallmedizin.

[3] Valley T.S., Noritomi D.T. (2020). ICU beds: Less is more? Yes. Intensive Care Med..

[4] 220310-Qualitaetsindikatoren-Intensivmedizin-divi-Peer-Review. https://www.divi.de/publikationen/alle-publikationen/service/peer-review?start=20.

[5] Markewitz A., Waydhas C., Riessen R., van den Hooven T. (2023). DIVI-Empfehlung zu Struktur und Ausstattung von Intensivstationen. Z. Für Herz-Thorax- Und Gefäßchirurgie.

[6] Hanson C.W., Durbin C.G., Maccioli G.A., Deutschman C.S., Sladen R.N., Pronovost P.J., Gattinoni L. (2001). The anesthesiologist in critical care medicine: Past, present, and future. Anesthesiology.

[7] Accreditation Council for Graduate Medical Education (2012). ACGME Program Requirements for Graduate Medical Education in Surgical Critical Care.

[8] Accreditation Council for Graduate Medical Education (2014). ACGME Program Requirements for Graduate Medical Education in Anesthesiology Critical Care Medicine.

[9] Zacharowski K., Filipescu D., Pelosi P., Åkeson J., Bubenek S., Gregoretti C., Sander M., de Robertis E. (2022). Intensive care medicine in Europe: Perspectives from the European Society of Anaesthesiology and Intensive Care. Eur. J. Anaesthesiol..

[10] Reisner-Sénélar L. (2011). The birth of intensive care medicine: Björn Ibsen’s records. Intensive Care Med..

[11] van Ackern K., Schwarz W., Striebel J., Schüttler J. (2003). 50 Jahre Deutsche Gesellschaft für Anästhesiologie und Intensivmedizin. 50 Jahre Deutsche Gesellschaft für Anästhesiologie und Intensivmedizin: Tradition & Innovation.

[12] Klar E., Püschel A., Schiffmann L., Pertschy A. (2009). Rolle der Intensivmedizin bei frühen postoperativen Komplikationen. Chirurg.

[13] Park C.A., Mcgwin G., Smith D.R., May A.K., Melton S.M., Taylor A.J., Rue L.W. (2001). Trauma-Specific Intensive Care Units Can be Cost Effective and Contribute to Reduced Hospital Length of Stay. Am. Surg..

[14] Giannoudis P.V. (2002). When is the safest time to undertake secondary definitive fracture stabilization procedures in multiply injured patients who were initially managed using a strategy of primary temporary skeletal fixation. J. Trauma.

[15] Lee J., Iqbal S., Gursahaney A., Nouh T., Khwaja K. (2013). Medicine versus surgery/anesthesiology intensivists: A retrospective review and comparison of outcomes in a mixed medical-surgical-trauma ICU. Can. J. Surg..

[16] Lott J.P., Iwashyna T.J., Christie J.D., Asch D.A., Kramer A.A., Kahn J.M. (2009). Critical illness outcomes in specialty versus general intensive care units. Am. J. Respir. Crit. Care Med..

[17] Diringer M.N., Edwards D.F. (2001). Admission to a neurologic/neurosurgical intensive care unit is associated with reduced mortality rate after intracerebral hemorrhage. Crit. Care Med..

[18] Lombardo S., Scalea T., Sperry J., Coimbra R., Vercruysse G., Enniss T., Jurkovich G.J., Nirula R. (2017). Neuro, trauma, or med/surg intensive care unit: Does it matter where multiple injuries patients with traumatic brain injury are admitted? Secondary analysis of the American Association for the Surgery of Trauma Multi-Institutional Trials Committee decompressive craniectomy study. J. Trauma Acute Care Surg..

[19] Sarikouch S., Haverich A. (2011). Improving surgeons impact on intensive care medicine. Zentralblatt Fur Chir..

[20] Waydhas C., Seekamp A., Sturm J.A. (2006). Intensivmedizin aus Sicht des Unfallchirurgen. Chirurg.

[21] Abbenbroek B., Duffield C.M., Elliott D. (2014). The intensive care unit volume–mortality relationship, is bigger better? An integrative literature review. Aust. Crit. Care.

[22] Glance L., Li Y., Osler T., Dick A., Mukamel D. (2006). Impact of patient volume on the mortality rate of adult intensive care unit patients. Crit. Care Med..

[23] Schreiter D., Saeger H. (2011). Unter welchen Voraussetzungen ist das Konzept einer interdisziplinären operativen Intensivmedizin praktikabel?. Zentralblatt Für Chir..

[24] Neuhaus P. (1999). Chirurgische Intensivmedizin aus der Sicht des Chirurgen. Chirurg.

